# Optimization of fermentation upstream parameters and immobilization of *Corynebacterium glutamicum* MH 20-22 B cells to enhance the production of l-lysine

**DOI:** 10.1007/s13205-014-0252-7

**Published:** 2014-09-27

**Authors:** Meerza Abdul Razak, Buddolla Viswanath

**Affiliations:** 1 Natco Pharma Limited, Natco House, Road No. 2, Banjara Hills, Hyderabad, 500 034 India; 2 Department of Virology, Sri Venkateswara University, Tirupati, 517502 AP India

**Keywords:** l-Lysine, *C. glutamicum*, Immobilization, Upstream process parameters

## Abstract

l-Lysine is an essential amino acid with high commercial importance, as it has to be available in sufficient quantities in animal and human feeds to meet their nutritional requirement. As there is constant increase in l-lysine demand every year, to meet the increasing demand it is necessary to produce l-lysine in large scale. Generally, l-lysine is produced by batch fermentation. In the present investigation, different fermentation process parameters such as fermentation time, pH, temperature, glucose concentration, airflow rate and aeration rate were studied to optimize the production of l-lysine by *Corynebacterium glutamicum* MH 20-22 B in a 5 L laboratory-scale stirred tank bioreactor. A comparative study of l-lysine production with free cells and immobilized cells of *C. glutamicum* MH 20-22 B was also investigated to determine whether free cells or immobilized cells were advantageous for production of l-lysine. In this way, optimized fermentation upstream parameters which produced the maximum yield of l-lysine were developed and it was also concluded from the present study that immobilized cells of *C. glutamicum* MH 20-22 B were more advantageous for l-lysine production as they yield more l-lysine compared to free cells of *C. glutamicum* MH 20-22 B. It was observed in the present study that the optimum values of fermentation time, pH, temperature, glucose concentration, airflow rate and aeration rate were 96 h, 7.5, 30 °C, 90 g/l, 1.0 vvm and 200 rpm, respectively, by immobilized cells, whereas in case of free cells the optimum values were 72 h, 7.5, 30 °C, 80 g/l, 1.25 vvm and 300 rpm. Immobilized *C. glutamicum* MH 20-22 B cells exhibited greater l-lysine production of 31.58 g/l than free cells which produced 26.34 g/l of l-lysine.

## Introduction

Amino acids have long played a vital role in both human and animal nutrition and health maintenance (Bercovici and Fuller 2007). With the advent of new applications and the steadily growing market demand of amino acids, their production technology has made an enormous progress during the second half of the twentieth century. Fermentation and enzymatic catalysis are the two biotechnological processes which posses both ecological and economical advantage and are the main reason for the spectacular growth of the amino acid industry. The feed amino acids l-lysine, dl-methionine, l-threonine, and l-tryptophan contribute the largest share (56 %) of the total amino acid market, approximately US$ 4.5 billion (Leuchtenberger et al. 2005). Low-cost fermentation processes have been developed for different kinds of amino acids and the current rapid progress in biotechnology, including biochemical engineering technology, strain improvement along with downstream processing, indicates that the fermentation process is the key role in the amino acid industry. Over a period of time, fermentation technology has played a vital role and recently amino acids produced by fermentation processes are the main products of biotechnology in value and volume (Ikeda 2003).

l-Lysine (C_6_H_14_N_2_O_2_; MW 146.19) is one of the essential, commercial and important amino acid which is produced on large scale by the amino acid industry. l-lysine is generally produced in purity higher than 98.5 % in stable and non-hygroscopic hydrochlorinated form (Fechter et al. 1997). l-Lysine has a number of applications in the food, pharmaceutical, feed milling and cosmetics industries, and its current price is approximately US$ 3–4/kg. The production costs of l-lysine have been lowered because of continuous optimization of the fermentation process. 80 % of lysine in the world market is synthesized by microbial fermentation and the remaining 20 % by chemical synthesis. Thus, the scope for l-lysine production in the amino acid industry is more because of its increasing market demand (Anastassiadis 2007). The raw materials used in the fermentation process for the production of l-lysine are biologically and naturally available, and the by-products produced during l-lysine fermentation are non-toxic and have high commercial value. The microbes separated from the broth contain more than 50 % of proteins, which are used in animal feed. Organic and inorganic nitrogen compounds, phosphorus compounds and potassium salts, which could be used as fertilizers, are also produced in the spent broth during l-lysine production (Anastassiadis 2007).

Microbial strains such as *Brevibacterium lactofermentum* (Fechter et al. 1997), *Brevibacterium flavum* (Ikeda 2003) and *C. glutamicum* (Anastassiadis 2007) have been used for the past 50 years for large-scale production of l-lysine, but the sole production organism for l-lysine is *C. glutamicum* and its subspecies. l-Lysine is produced by an aerobic fermentation process using *C. glutamicum,* which is a rod-shaped, fast-growing, non-sporulating, Gram-positive and nonpathogenic coryneform bacterium (Kinoshita et al. 1957; Udaka 1960). The ability of *C. glutamicum* to produce other amino acids, such as l-threonine (Nakayama and Kase 1974), l-methionine (Kase and Nakayama 1975), l-serine (Eggeling 2007), l-histidine (Araki et al. 1974), l-valine (Ruklisha et al. 2007), l-tryptophan (Ikeda 2006), l-phenylalanine and l-tyrosine (Ikeda and Katsumata 1992), l-leucine (Patek 2007) and l-isoleucine (Guillout et al. 2002), has made it an important organism in industrial biotechnology. Large-scale production of l-lysine by fermentation with *C. glutamicum* started in 1958 at Kyowa Hakko’s plant in Japan. The biotechnological production of l-lysine has been constantly improved by fermentation process optimization and strain improvement (Pfefferle et al. 2003). Fermentation time, pH, temperature, glucose concentration, airflow rate and aeration rate are the most important factors in fermentation processes. However, there have been very few reports on fermentation process optimization. Hence, the main objective of this paper is to study l-lysine production by fermentation of free and immobilized cells using different culture parameters.

## Materials and methods

*C. glutamicum* MH 20-22 B, which is a leucine auxotroph, was employed throughout this study. *C. glutamicum* MH 20-22 B was donated by Professor Eggeling, Biotechnology Institute, Julich, Germany. It was cultured on agar slopes using nutrient agar medium containing peptone (5 g), beef extract (3 g), NaCl (5 g), agar (15 g) and distilled water (1,000 ml). The pH was maintained at 7.0. The composition of the media used in the fermentation process was as follows:

### Rich medium

Glucose (10 g), yeast extract (10 g), peptone (10 g), NaCl (2.5 g) and distilled water (1.0 L); the pH was maintained at 7.0.

### Inoculum medium

CaCl_2_·2H_2_O (1 g), (NH_4_)·2SO_4_ (30 g), MgSO_4_·7H_2_O (0.4 g), NaCl (0.05 g), MnSO_4_·H_2_O (0.0076 g), FeSO_4_·7H_2_O (0.001 g), KH_2_PO_4_ (0.5 g), K_2_HPO_4_ (0.5 g), Urea (2 g), yeast extract (1 g), peptone (1 g), d-glucose (10 g), thiamine (0.2 mg), d-biotin (0.5 mg) and distilled water (1.0 L); the pH was maintained at 7.0.

### Fermentation medium

CaCl_2_·2H_2_O (1 g), (NH_4_)·2SO_4_ (30 g), MgSO_4_·7H_2_O (0.4 g), NaCl (0.05 g), MnSO_4_·H_2_O (0.0076 g), FeSO_4_·7H_2_O (0.001 g), KH_2_PO_4_ (1 g), K_2_HPO_4_ (1 g), urea (2 g), yeast extract (1.5 g), peptone (2 g), d-glucose (150 g), thiamine (0.2 mg), d-biotin (0.5 mg), l-serine (0.1 mg) and distilled water (1.0 L); the pH was maintained at 7.0.

### Fermentation procedure

*C. glutamicum* MH 20-22 B cells were cultured on a nutrient agar strain maintenance plate at 30 °C for 24 h and inoculated into 20 ml of rich medium in a 250 ml conical flask. After 24 h of cultivation in an orbital shaker with 120 rpm at 30 °C, the seed broth was transferred to 180 ml of inoculum media in an Erlenmeyer flask and maintained in an orbital shaker for 40 h at 120 rpm and 30 °C. This was then used to inoculate the fermentation medium in the stirred tank bioreactor. Batch fermentation experiments were carried out at different parameters to optimize the upstream parameters for free cells and immobilized cells.

### Immobilization method

#### Growth medium composition

Glucose (2 g), beef extract (1 g), Bacto Peptone (1 g), NaCl (0.25 g), agar (2 g) and distilled water (100 ml) were maintained at pH 7.0. Agar slants of *C. glutamicum* MH 20-22 B which were grown for 24 h were used to inoculate 50 ml of growth medium (and kept on shaker for 48 h (150 rpm) at 30 °C. 100 ml of 72 h-old culture was used to prepare immobilized beads of calcium alginate. 15 % volume of beads were employed throughout this study. Immobilization of *C. glutamicum* MH 20-22 B cells was done in strict aseptic conditions. Gluteraldehyde entrapment method using cross-linked calcium alginate was used to immobilize *C. glutamicum* MH 20-22 B cells (Jetty et al. 2005; Marek et al. 1985). 100 µl gluteraldehyde and 3 % sodium alginate were thoroughly mixed with 0.06 % cells on dry cell weight basis (DCW) (w/v) to get uniform suspension. This uniform suspension was transferred into 0.2 M CaCl_2_ solution using a peristaltic pump through a cut micropipette tip (or) orifice. The curing of the formed beads was done by incubating in 0.2 M CaCl_2_ solution for 24 h. The beads were washed twice with sterile saline solution (0.9 % NaCl solution (w/v)) and preserved at 4 °C in saline solution for further use.

#### Sterilization of the stirred tank bioreactor

Initially, all reactor parts were separated. All parts were washed with distilled water thoroughly and again with acetone before sterilization. After washing with distilled water, all the above parts were wrapped with aluminum foil before placing in the autoclave. All these accessories were kept in the autoclave, which was operated at a temperature of 120 °C and a pressure of 15 psi for a period of 20 min.

### Fermentation studies

Batch or fed-batch processes are employed for the commercial production of amino acids. In batch operations all of the nutrients are added at the beginning. Moreover, in batch fermentations microorganisms grows until one or more of essential nutrients get exhausted or until fermentation conditions such as oxygen limitation, pH decrease and product inhibition become unfavorable. In this present investigation, fermentation experiments with free cells and immobilized cells of *C. glutamicum* MH 20-22 B were conducted with different fermentation parameters at batch mode using a sterilized continuous stirred tank bioreactor of 5 L capacity. For experiments with immobilized cells of *C. glutamicum*, a fermentation medium containing 60 g of immobilized beads (15 %) was used throughout the experiments. Beads containing 3.0–3.5 mm range of diameter were used. Different parameters with different variations were studied, and variations in the particular parameter were made as per the previous experiments and literature.

### Analytical methods

l-Lysine in the supernatant fluid was quantitatively estimated by the acidic ninhydrin method (Chinard 1952). Throughout the study, the glucose concentration was estimated by the anthrone method (Morris 1948; Neish 1952). The dry cell weight (Biomass) was determined (Kim et al. 1981).

## Results and discussions

The present report describes the optimization of fermentation parameters for l-lysine production by *C. glutamicum* MH 20-22 B. The continuous batch fermentation described in this study also facilitated the analysis of the various parameters affecting l-lysine fermentation. Earlier, many investigators have reported about multistage continuous fermentation processes and their difficulties in running continuous l-lysine production systems (Becker 1982; Michalski et al. 1984). To determine the suitable conditions for l-lysine production by free and immobilized cells of *C. glutamicum* in a stirred tank bioreactor, the parameters investigated are l-lysine concentration, biomass concentration, residual glucose concentration and glucose utilized with variations in fermentation time, temperature, pH, substrate concentration (glucose), aeration and agitation rate.

### Effect of fermentation time on l-lysine production by free cells

**Fig. 1:**
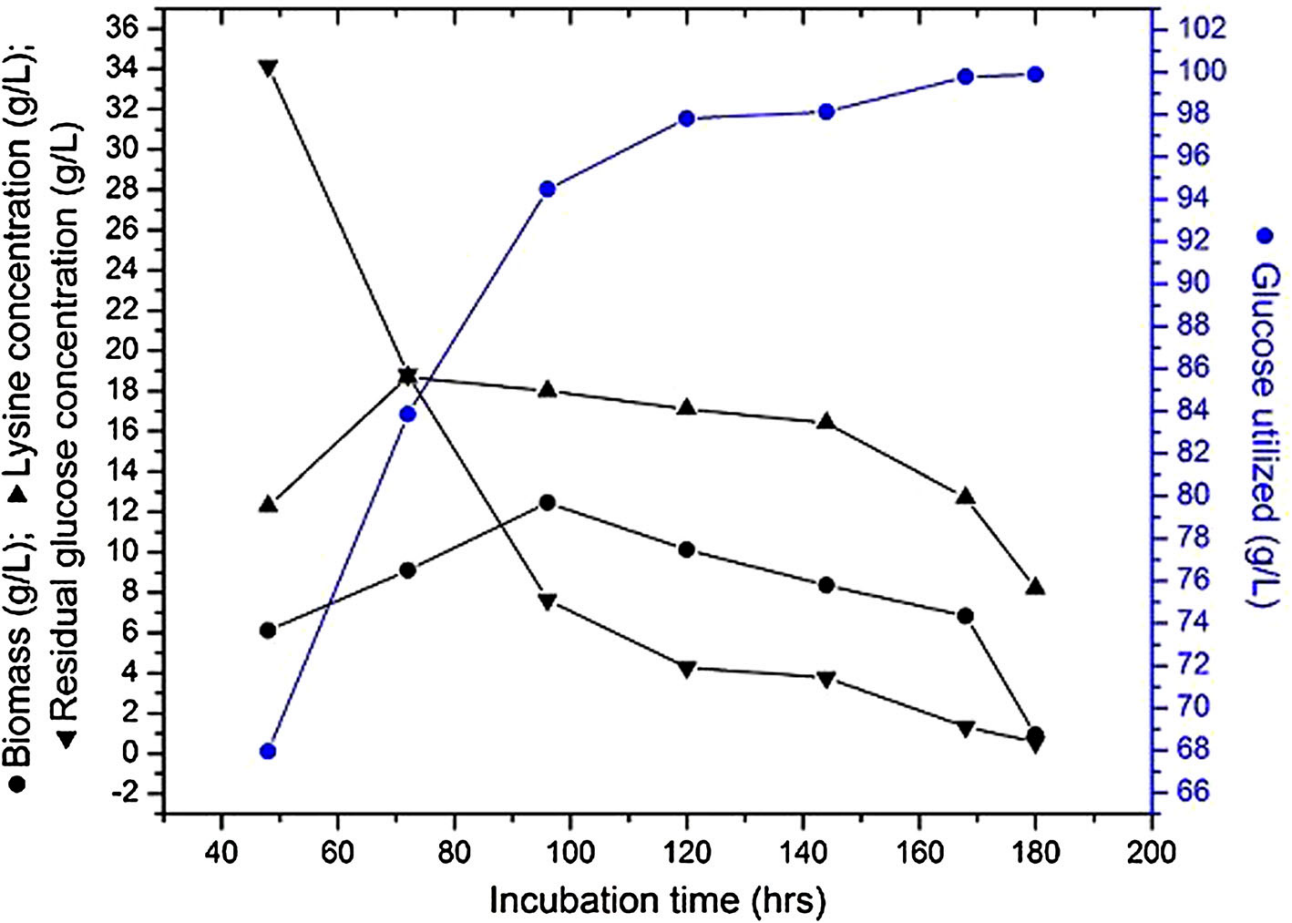
Effect of fermentation time on l-lysine production by free cells

The effect of fermentation time on l-lysine production by free cells of *C. glutamicum* MH 20-22 B was studied maintaining the operating conditions as temperature of 28 °C, pH 7.0, air flow rate 1.5 vvm, agitation rate 300 rpm and glucose concentration 100 (g/l). It was analyzed from Fig. 1 that as the fermentation time increased, the concentration of the residual glucose decreased from 34.13 to 18.80 g/l between 48 and 72 h and the product concentration also increased from 12.3 to 18.7 g/l with biomass concentration from 6.11 to 9.1 g/l. Similar studies were carried out by Ekwealor and Obeta (2005) for the production of lysine using *Bacillus megaterium* free cells. As the fermentation proceeded further, the glucose consumption and l-lysine production increased and reached the maximum at 72 h, with increased sugar utilization rate and maximum l-lysine concentration of 18.7 g/l being observed (Fig. 1). After 72 h, the maximum product achieved a downward trend and l-lysine concentration and yields decreased, in spite of the increase in fermentation time due to less availability of nutrients. So a fermentation time of 72 h was most suitable for the production of l-lysine using *C. glutamicum* MH 20-22 B free cells. Hadj Sassi et al. (1988) studied the yield of l-lysine (Yp/s) by *C. glutamicum* in a fed-batch reactor using free cells, and the maximum yield was 0.21 (g/g).

### Effect of temperature on l-lysine production by free cells

The growth rate of bacterial microorganisms is strongly dependent on temperature during fermentation which changes the whole metabolism. In this present study, an attempt has been made to study the effect of different temperature ranges (27–32 °C) on l-lysine production carried out in a stirred tank reactor. The temperature profile on l-lysine production was done using *C. glutamicum* MH 20-22 B free cells under operating conditions such as fermentation time of 72 h, pH 7.0, air flow rate 1.5 vvm, agitation rate 300 rpm and glucose concentration 100 g/l. Any change in the temperature can alter the substrate utilization rate of microorganism, which leads to unbalanced nutrients in the medium with respect to the growth rate of the *C. glutamicum* cells. If any of the crucial nutrients is exhausted soon or unused, this can make the growth in culture from balanced to unbalanced and results in performance change. At 30 °C, maximum l-lysine concentration of 21.31 (g/l) was obtained along with 18.24 g/l of maximum biomass (Table 1). Glucose utilization was also high at 30 °C. An increase in temperature resulted in decreased productivity, which suggested that a small increase in temperature has a profound effect on cellular activities, such as repression of metabolic enzymes. Hilliger et al. (1984) observed the influence of temperature as one of the major fermentation parameters on growth and l-lysine formation by *C. glutamicum*. They studied l-lysine production at different temperatures which showed that biomass and l-lysine concentration was high at 30 °C, whereby temperatures exceeding 32 °C reduced the specific l-lysine formation rate and the substrate conversion yield coefficient. Hence, a temperature of 30 °C was optimum temperature for l-lysine fermentation by both free and immobilized cells of *C. glutamicum* MH 20-22 B.

**Table 1:** Effect of temperature on l-lysine production by free cells

Sl. no.	Temperature (^o^C)	Lysine conc. (*p*) (g/l)	Biomass (*x*) (g/l)	Residual glucose conc. (g/l)	Glucose utilized (*s*) (g/l)
1	27	14.6	13.35	8.54	93.47
2	28	18.4	14.78	5.21	94.78
3	29	19.6	15.67	4.32	97.77
4	30	21.31	18.24	2.65	99.34
5	31	19.7	16.52	3.53	98.56
6	32	17.9	14.43	5.25	96.84

### Effect of pH on l-lysine production by free cells

In microbial fermentations, pH is a very important and strongly influencing factor. Basic compounds, such as ammonium hydroxide, potassium hydroxide, sodium hydroxide, calcium carbonate, ammonia and gaseous ammonia and urea, inorganic acid compounds such as sulfuric or phosphoric acid and organic acids are utilized for controlling pH in l-lysine cultures ranging from 5.0 to 9.0 (Nakamura et al. 2000). The effect of pH was found to be a very significant parameter in l-lysine yield. The pH effect on l-lysine production was investigated under operating conditions such as fermentation time of 72 h, temperature of 30 °C, air flow rate of 1.5 vvm, agitation rate of 300 rpm and glucose concentration rate of 100 g/l. From Table 2, it may be analyzed that the optimum pH for *C. glutamicum* MH 20-22 B free cells is 7.5, with the maximum lysine concentration of 22.58 (g/l). At 7.5 pH the biomass production was also high at 17.85 g/l. Broer et al. (1993) also reported that the optimum pH for maximum velocity transport of *C. glutamicum* was 7.4–7.8. To maintain optimal pH, reagents such as calcium carbonate must be added to the culture medium at the beginning of fermentation. Thus, calcium carbonate was used as an internal neutralizing agent. Though the pH of the fermenter was automatically controlled by ammonia water, a small amount of CaCO_3_ must be added (Wang et al. 1991). It eliminates the lag phase of cell growth, thereby shortening fermentation time. Therefore 7.0 was considered as an optimum pH for all the experiments.

**Table 2:** Effect of pH on l-lysine production by free cells

Sl. no	pH	Lysine conc. (*p*) (g/l)	Biomass (*x*) (g/l)	Residual glucose conc. (g/l)	Glucose utilized (*s*) (g/l)
1	6	12.3	13.62	8.89	93.02
2	6.5	14.4	14.53	5.63	96.46
3	7	19.42	14.87	2.53	97.66
4	7.5	22.58	17.85	0.52	99.27
5	8	18.57	13.34	3.29	98.08
6	8.5	16.52	12.98	4.53	97.56

### Effect of glucose concentration on l-lysine production by free cells

In fermentation, the cell concentration and substrate concentration play major roles in the overall performance of the process for l-lysine production. The cells use available substrate immediately if the process conditions in the reactor are favorable. The viability and growth of the cells are very much dependent on the substrate availability in the reactor. l-Lysine-producing bacteria can utilize various carbon sources, such as glucose, fructose, sucrose and maltose. The effect of substrate on the l-lysine yield was expressed on the basis of lysine produced per unit substrate consumed. The effect of glucose concentration on l-lysine production by free cells of *C. glutamicum* MH 20-22 B was studied by carrying fermentation under conditions of pH 7.5, fermentation time 72 h, temperature 30 °C, air flow rate 1.5 vvm, agitation rate 300 rpm and different ranges of glucose concentrations of 70, 80, 90, 100, 110 and 120 (g/l).

Glucose concentration on l-lysine production was investigated by Hirose and Shibai (1985) and it was found that higher concentration of glucose inhibited bacterial growth along with low yield. For this purpose, the effects of different concentrations of glucose on l-lysine production were examined. Different l-lysine batch fermentations were conducted at different initial glucose concentrations. From different batches, it was apparent that lysine production was cell growth associated and this was clearly confirmed. From Table 3 the maximum l-lysine concentration of 20.1 (g/l) is observed at glucose concentration of 80 g/l for *C. glutamicum* MH 20-22 B free cells and any excessive substrate concentration present in the fermentation broth leads to reduction in the product concentration because of substrate inhibition. (Hadj Sassi et al. 1988) reported that the initial concentration of glucose influenced the production of l-lysine by *Corynebacterium* Sp. in batch culture and found that the specific production rate was obtained at 65 g/l of glucose. However, it was observed that pH and substrate concentration had significant effect in comparison to temperature.

**Table 3:** Effect of glucose concentration on l-lysine production by free cells

Sl. no.	Glucose concentration (g/l)	Lysine conc. (*p*) (g/l)	Biomass (*x*) (g/l)	Residual glucose conc. (g/l)	Glucose utilized (*s*) (g/l)
1	70	14.7	5.13	3.62	68.47
2	80	20.1	9.35	4.54	67.67
3	90	19.89	16.59	6.08	85.29
4	100	18.1	17.23	10.57	91.52
5	110	13.9	17.98	14.64	97.45
6	120	11.5	18.32	18.25	99.48

### Effect of airflow rate on l-lysine production by free cells

The air supply in submerged cultures is known to have an important influence on microbial production of amino acids (Akashi et al. 1979). Thus, oxygen has been shown to play an important and crucial role in the regulation of both intermediary metabolism and biomass formation coupled with alteration of l-lysine synthesis and with a change of l-lysine yield, respectively. To find the optimum value of air flow rate for free cells of *C. glutamicum* MH 20-22 B, experimental studies were conducted in a batch-stirred tank reactor in the bioreactor under conditions of pH 7.5, fermentation time 72 h, temperature 30 °C, agitation rate 300 rpm, glucose concentration 80 g/l and the aeration rate maintained at different ranges from 0.25 to 1.5. Table 4 shows the characteristic batches of l-lysine fermentation at a range of 0.25–1.5 volume air per volume of medium per minute (vvm). The best air flow rate for l-lysine production by free cells was 1.25 vvm, the product concentration was 20.54 g/l and the biomass productivity was 19.34 g/l. Glucose utilized was 80.66 g/l at 1.25 vvm. So 1.25 vvm was considered as the optimum airflow rate for *C. glutamicum* MH 20-22 B free cells. Wang et al. (1991) performed fermentation of l-lysine on a rotary shaker at 200 rpm and suggested that the optimum air flow rate of l-lysine production was 1.0 vvm or above 1.0 vvm, approximately.

**Table 4:** Effect of airflow rate on l-lysine production by free cells

Sl. no.	Air flow rate (vvm)	Lysine conc. (*p*) (g/l)	Biomass (*x*) (g/l)	Residual glucose conc. (g/l)	Glucose utilized (*s*) (g/l)
1	0.25	11.54	09.76	7.31	74.96
2	0.5	15.47	12.37	5.35	76.74
3	0.75	16.26	16.54	4.47	77.62
4	1	19.53	18.28	3.33	78.76
5	1.25	20.54	19.34	1.57	80.66
6	1.5	18.43	17.74	2.56	78.67

### Effect of agitation rate on l-lysine production by free cells

Agitation and aeration in a stirred tank bioreactor always cause foaming. Excess foaming forces the broth out of the bioreactor and contaminates the system quickly. Optimum aeration must be maintained, as agitation minimizes foaming and maximizes the specific production rate. In the bioreactor, the fermentation medium was agitated to provide homogeneity across the vessel. l-lysine production at different agitation rates of 100, 150, 200, 300, 350 and 400 rpm and under different conditions such as pH 7.5, fermentation time 72 h, temperature 30 °C, aeration rate 1.25 vvm and glucose concentration 80 g/l was examined to find out the optimum agitation rate for l-lysine production by free cells of *C. glutamicum* MH 20-22 B. From Table 5, we can observe that the maximum l-lysine concentration is 26.34 g/l and the biomass is 17.13 g/l at 300 rpm. Therefore, the optimum agitation rate for l-lysine production was 300 rpm for free cells. Shah et al. (2002) studied the influence of dilution in the range between 50 and 300 rpm and concluded that after 200 rpm l-lysine production was levelled off.

**Table 5:** Effect of agitation rate on l-lysine production by free cells

Sl. no.	Agitation rate (rpm)	Lysine conc. (*p*) (g/l)	Biomass (*x*) (g/l)	Residual glucose conc. (g/l)	Glucose utilized (*s*) (g/l)
1	100	19.58	11.78	7.54	94.55
2	150	22.73	14.65	6.50	95.59
3	200	23.37	15.98	4.23	97.86
4	250	25.95	16.35	3.56	98.53
5	300	26.34	17.13	2.51	99.58
6	350	23.85	14.43	3.48	98.16
7	400	22.39	15.72	5.67	96.32

### Production of l-lysine by immobilized cells of *C. Glutamicum*

#### Effect of fermentation time on l-lysine production by immobilized cells

**Fig. 2:**
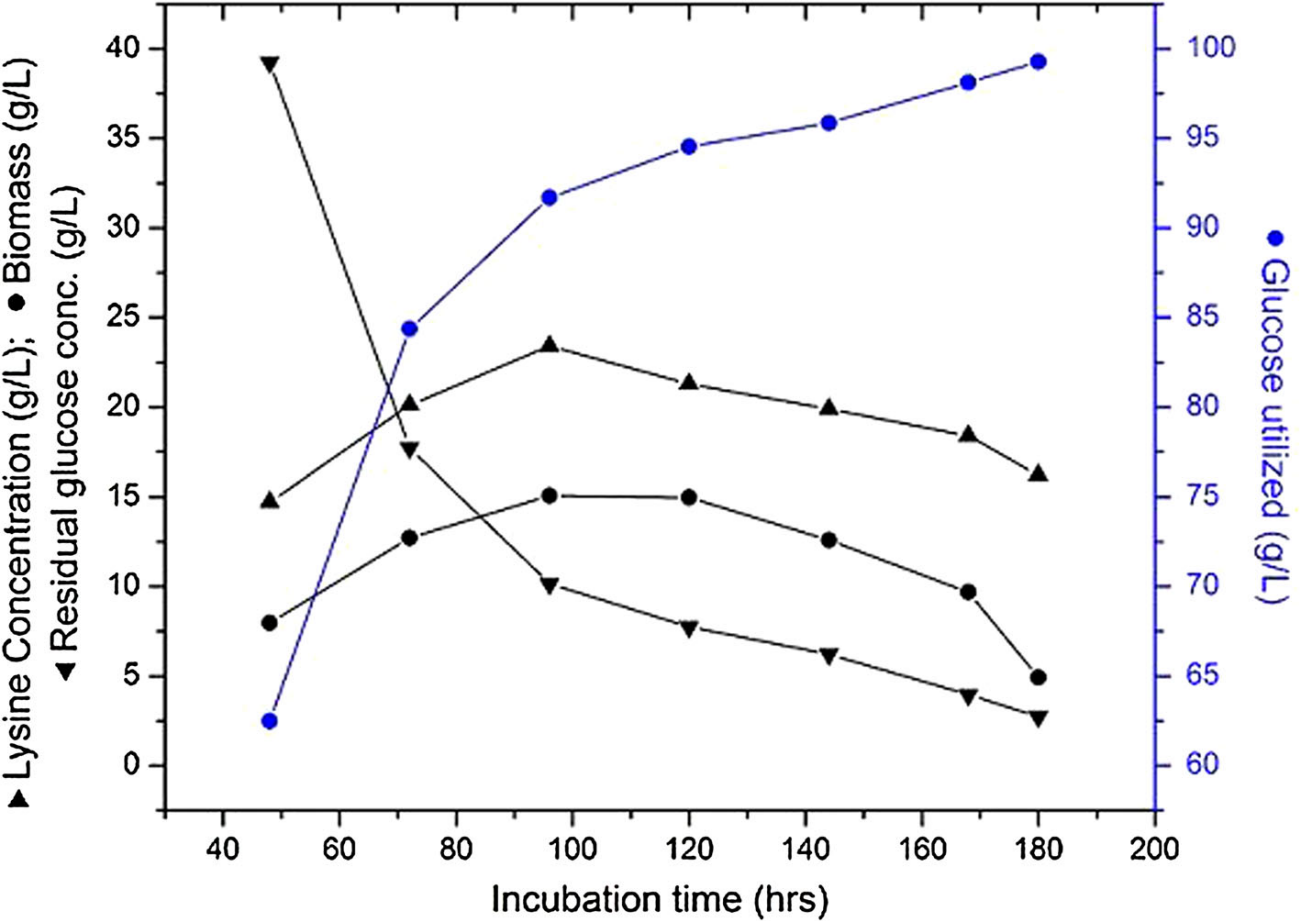
Effect of fermentation time on l-lysine production by immobilized cells

Studies on the effect of fermentation time on l-lysine production by immobilized *C. glutamicum* MH 20-22 B cells were carried out under different fermentation conditions, such as temperature 28 °C, pH 7.0, air flow rate 1.5 vvm, agitation rate 300 rpm and glucose concentration 100 (g/l). From Fig. 2, it may be analyzed that l-lysine concentration is relatively low over the first 2 (48 h) days. l-lysine accumulation was 20.1 g/l at 72 h and later increased to 23.4 g/l at 96 h. The biomass concentration was 15.07 g/l at 96 h. After 96 h, an important observation was the lower glucose concentration of 10.15 g/l, which further decreased as the fermentation process proceeded. l-lysine production decreased due to the depletion of nutrients in the fermentation medium. The results presented in Fig. 2 show that l-lysine production is good at 96 h, which is the best fermentation time for the production of l-lysine by immobilized cells of *C. glutamicum* MH 20-22 B.

#### Effect of temperature on l-lysine production by immobilized cells

The effect of temperature on l-lysine production by immobilized cells of *C. glutamicum* MH 20-22 B was studied under different operating conditions such as fermentation time of 96 h, pH 7.0, air flow rate 1.5 vvm, agitation rate 300 rpm and glucose concentration 100 g/l. The results from Table 6 show that l-lysine concentration of 24.45 g/l, biomass of 19.45 g/l and glucose utilization of 95.67 g/l, which were maximum compared to all temperatures, were observed at 30 °C. Therefore, 30 °C temperature was most suitable for the production of l-lysine by *C. glutamicum* MH 20-22 B. The downtrend of residual glucose as the temperature increases can be seen in Table 6. After 30 °C, the l-lysine and biomass concentrations were decreased. Therefore, 30 °C was the optimum temperature for l-lysine production by immobilized cells of *C. glutamicum* MH 20-22 B.

**Table 6:** Effect of temperature on l-lysine production by immobilized cells

Sl. no.	Temperature (^o^C)	Lysine conc. (*p*) (g/l)	Biomass (*x*) (g/l)	Residual glucose conc. (g/l)	Glucose utilized (*s*) (g/l)
1	27	17.89	15.15	12.33	89.76
2	28	21.74	15.99	9.64	92.45
3	29	22.33	16.79	8.61	93.48
4	30	24.45	19.45	6.42	95.67
5	31	23.20	18.82	4.66	97.43
6	32	21.74	16.44	2.68	99.41

#### Effect of pH on l-lysine production by immobilized cells

The growth rate of microorganism is usually very sensitive to variation in pH. Furthermore, the process of growth changes the pH of the medium; therefore, the effect of different pH values on l-lysine production was examined in a stirred tank bioreactor. The pH profile of immobilized cells of *C. glutamicum* MH 20-22 B was studied by carrying fermentation under conditions such as fermentation time of 96 h, temperature 30 °C, air flow rate 1.5 vvm, agitation rate 300 rpm and glucose concentration 100 g/l. The pH ranges were maintained from 6 to 8.5. The different pH values (6–8.5) of the production medium were adjusted with 2 M HCl and 1 M NaOH. Table 7 shows that the maximum l-lysine concentration of 25.62 (g/l) and biomass of 20.42 (g/l) were obtained at pH of 7.5 for immobilized cells of *C. glutamicum* MH 20-22 B. Table 7 also shows that as the pH increases, the residual glucose concentration decreases. When compared with all pH ranges, 7.5 was the best pH for l-lysine production with immobilized cells.

**Table 7:** Effect of pH on l-lysine production by immobilized cells

Sl. no	pH	Lysine conc. (*p*) (g/l)	Biomass (*x*) (g/l)	Residual glucose conc. (g/l)	Glucose utilized (*s*) (g/l)
1	6	14.69	15.47	12.86	89.23
2	6.5	19.9	18.68	10.44	91.65
3	7	21.78	19.33	7.34	94.75
4	7.5	25.62	20.42	5.60	96.49
5	8	21.71	17.53	4.41	97.68
6	8.5	19.7	15.62	2.68	99.41

#### Effect of glucose concentration on l-lysine production by immobilized cells

In the bioreactor, the cell concentration and glucose concentration play a major role in the overall performance of the process for l-lysine production. The cells use available glucose immediately if the process conditions in the reactor are favorable. The viability and growth of the cell are very much dependent on the glucose availability in the bioreactor. For this purpose, different glucose concentrations [70, 80, 90, 100, 110 and 120 (g/l)] were employed under different conditions such as pH 7.5, fermentation time 96 h, temperature 30 °C, air flow rate 1.5 vvm and agitation rate 300 rpm to determine its effect on l-lysine production. The maximum concentrations of l-lysine of 26.59 g/l and biomass of 19.68 g/l were obtained at 90 g/l glucose concentration in spite of low residual concentration (Table 8). Glucose concentration of 90 g/l was found to be effective in terms of l-lysine production with immobilized cells of *C. glutamicum* MH 20-22 B.

**Table 8:** Effect of glucose concentration on l-lysine production by immobilized cells

Sl. no	Substrate conc. (g/l)	Lysine conc. (*p*) (g/l)	Biomass (*x*) (g/l)	Residual glucose conc. (g/l)	Glucose utilized (*s*) (g/l)
1	70	18.5	7.93	4.61	67.48
2	80	21.10	11.38	6.66	75.43
3	90	26.59	19.68	7.47	84.62
4	100	23.7	18.64	12.33	89.76
5	110	20.10	17.46	16.85	97.24
6	120	17.5	16.64	20.66	99.34

#### Effect of aeration rate on l-lysine production by immobilized cells

The oxygen supply is greatly vital for the fermentation process. Oxygen, an extremely important nutrient, is usually supplied to the reactor by an air pump. Experimental studies were conducted in a batch-stirred tank reactor to find the optimum value of air flow rate for *C. glutamicum* MH 20-22 B in the bioreactor under conditions of pH 7.5, fermentation time 96 h, temperature 30 °C, agitation rate 300 rpm, glucose concentration 90 g/l and the aeration rate maintained at different ranges from 0.25 to 1.5. The maximum concentrations of l-lysine of 26.85 g/l and biomass of 20.93 g/l were obtained at an optimum air flow rate of 1.0 vvm for stirred tank bioreactor (Table 9). The maximum product concentration, biomass and glucose utilized were good at the flow rate of 1.0 vvm. Therefore, the optimum air flow rate for l-lysine production was found to be 1.0 vvm.

**Table 9:** Effect of aeration rate on l-lysine production by immobilized cells

Sl. no	Aeration rate (vvm)	Lysine conc. (*p*) (g/l)	Biomass (*x*) (g/l)	Residual glucose conc. (g/l)	Glucose utilized (*s*) (g/l)
1	0.25	16.97	11.39	12.41	78.68
2	0.5	19.68	14.95	10.35	81.56
3	0.75	21.56	19.09	6.96	85.04
4	1	26.85	20.93	5.26	86.47
5	1.25	25.97	18.37	3.53	88.56
6	1.5	24.44	16.78	3.02	88.97

#### Effect of agitation rate on l-lysine production by immobilized cells

Experimental studies to know the agitation profile of immobilized cells of *C. glutamicum* MH 20-22 B for better production of l-lysine were done by carrying out fermentation under different conditions such as pH 7.5, fermentation time 96 h, temperature 30 °C, aeration rate 1.0 vvm, glucose concentration 90 g/l and the agitation varied from ranges 100 to 400. In the bioreactor, a stirrer is present to maintain uniform composition throughout the vessel. Maximum l-lysine concentration and yield observed at 200 rpm were 31.58 g/l and biomass of 17.72 g/l, respectively (Table 10). Therefore, the optimum agitation rate for l-lysine production was 200 rpm for immobilized cells of *C. glutamicum* MH 20-22 B. Table 11 illustrates the optimized fermentation parameters for free cells and immobilized cells of *C. glutamicum* MH 20-22 B. The results obtained here indicate that l-lysine can be produced efficiently by immobilized growing *C. glutamicum* MH 20-22 B cells. By comparison with the results obtained with free cells, there is a significant improvement concerning the accumulation of l-lysine by immobilized growing cells.

**Table 10:** Effect of agitation rate on l-lysine production by immobilized cells

Sl. no	Agitation rate (rpm)	Lysine conc. (*p*) (g/l)	Biomass (*x*) (g/l)	Residual glucose conc. (g/l)	Glucose utilized (*s*) (g/l)
1	100	23.63	13.45	10.22	80.87
2	150	26.09	15.79	8.41	83.65
3	200	31.58	17.72	4.07	86.03
4	250	28.49	16.77	7.48	84.16
5	300	27.94	17.39	6.82	83.27
6	350	25.78	16.94	10.67	81.42
7	400	23.33	15.09	11.45	80.64

**Table 11:** Optimized fermentation parameters for free cells and immobilized cells of *C. glutamicum* MH 20-22 B

Fermentation conditions	Free cells of *C. glutamicum* MH 20-22 B	Immobilized cells of *C. glutamicum* MH 20-22 B
Fermentation time	72 h	96 h
pH	7.5	7.5
Temperature	30 °C	30 °C
Glucose concentration	80 g/l	90 g/l
Airflow rate	1.25 vvm	1.0 vvm
Aeration rate	300 rpm	200 rpm

## Conclusion

To improve the production rate of l-lysine, free and immobilized cell of *C. glutamicum* MH 20-22 B were analyzed under various process conditions. It was observed that the optimum values of fermentation time, pH, temperature, glucose concentration, airflow rate and aeration rate were 96 h, 7.5, 30 °C, 90 g/l, 1.0 vvm and 200 rpm, respectively, by immobilized cells. whereas in case of free cells the optimum values were 72 h, 7.5, 30 °C, 80 g/l, 1.25 vvm and 300 rpm. Immobilized *C. glutamicum* MH 20-22 B cells exhibited greater l-lysine production of 31.58 g/l than free cells, which produced 26.34 g/l of l-lysine. The results obtained here indicate that l-lysine can be produced efficiently by immobilized growing *C. glutamicum* MH 20-22 B cells by comparison with the results obtained with free cells in a stirred tank bioreactor.
